# A FRET-based off-on AIE nanoprobe enables instant and stain-free detection of hypoxic niches in tumor sections

**DOI:** 10.7150/thno.113038

**Published:** 2025-06-09

**Authors:** Chen Wang, Jun Shen, Muredili Muhetaer, Shenwu Zhang, Jin Sun, Zhonggui He, Yuequan Wang, Cong Luo

**Affiliations:** 1 Department of Pharmaceutics, Wuya College of Innovation, Shenyang Pharmaceutical University, Shenyang 110016, China.; 2 Joint International Research Laboratory of Intelligent Drug Delivery Systems of Ministry of Education, Shenyang Pharmaceutical University, Shenyang 110016, China.

**Keywords:** AIEgen, azobenzene, nanoassembly, FRET, tumor hypoxia detection

## Abstract

**Rationale:** Hypoxia is a critical hallmark of solid tumors, significantly influencing their diagnosis, treatment, and prognosis. Currently, instant and accurate detection of hypoxic niches in tumor sectioning remains a major challenge. Conventional tumor section staining methods lack reliability due to dynamic changes in hypoxic conditions during time-consuming sample processing.

**Methods:** Herein, we develop a FRET-based off-on AIE nanoprobe for instant and stain-free detection of tumor hypoxic niches following intravenous administration. A dimeric AIEgen (TNNT) is synthesized by conjugating two tetraphenylethene (TPE) molecules via azobenzene (Azo). TNNT self-assembles into stable nanoassemblies (NAs) with favorable drug delivery and tumor accumulation.

**Results:** Under normoxic conditions, fluorescence is quenched due to FRET between TPE and Azo, effectively "turning off" the AIE signal. Notably, the fluorescence recovers quickly following Azo cleavage under hypoxia *in vitro* and *in vivo*. Moreover, the AIE nanoprobe demonstrates an advantage over pimonidazole hydrochloride (HP3, a widely used hypoxia probe) in terms of *ex-vivo* hypoxia detection. Furthermore, it can also report the varying degrees of hypoxia in tumors of different sizes.

**Conclusions:** This study offers a practical tool for point-of-care tumor hypoxia detection and relevant pathological analysis.

## Introduction

Recently, the advancement of tumor microenvironment-responsive probes has emerged as a focal point in biomedical research, owing to their capacity to provide real-time and precise insights into pathological conditions 1-8. Among various tumor microenvironmental factors, hypoxia is particularly noteworthy. It arises from rapid tumor proliferation and the formation of abnormal blood vessels. These factors collectively lead to heterogeneous hypoxic niches in solid tumors 9, 10. The occurrence and extent of hypoxia throughout solid tumors are critical for understanding tumor progression and treatment resistance, underscoring the urgent need for precise and sensitive fluorescent probes capable of selectively detecting tumor hypoxia 11. Under hypoxic conditions, biological reductases (*e.g.,* azoreductase and nitroreductase) are overexpressed in tumors 12-14. Leveraging this pathological hallmark, various hypoxia-responsive fluorescent probes have been developed 15, 16. However, there are still significant challenges in their clinical translation. Most small-molecule probes undergo rapid systemic clearance following intravenous administration, leading to low tumor accumulation and limiting their ability to effectively target and detect hypoxic niches within solid tumors 17.

With continuous advancements in materials science and technology, biomedical nanotechnology enables more accurate and efficient approaches for cancer diagnosis and treatment 18-25. The use of functionalized nanocarriers significantly enhances stability, extends blood retention, and promotes tumor-targeting efficiency of therapeutic agents or imaging probes via mechanisms such as the enhanced permeability and retention (EPR) effect 26-28. However, although nanotechnology has substantially improved the delivery efficacy of conventional fluorescence probes, the imaging effect of nanoprobes is limited by the aggregation-caused quenching (ACQ) phenomenon after encapsulation into nanocarriers. In a landmark discovery in 2001, Tang and colleagues discovered aggregation-induced emission (AIE) phenomenon, which effectively addressed the ACQ effect and marked a breakthrough in the field of biological imaging and even phototherapy 29, 30. Notably, the aggregation-dependent fluorescence phenomenon of AIE fluorogens (AIEgens) is closely related to nanomedicine fabrication 31. Recently, numerous AIE-based nanoprobes have been developed for hypoxia detection 32. However, the clinical translation of these probes still faces significant challenges. Particularly, AIE nanoprobes continuously emit fluorescence in the aggregated state, limiting their luminescence specificity toward hypoxic environments 33. Moreover, the use of hypoxia-responsive nanocarrier materials in AIE nanoprobes is constrained by the lack of clinically approved excipients, further impeding their translational potential. Currently, point-of-care detection of tumor hypoxia is urgently desirable in clinical pathological analysis. Nevertheless, real-time *in vivo* detection of tumor hypoxia remains far from clinical practice, due to the rapid metabolic clearance and insufficient tumor accumulation of available probes. At present, post-section tumor staining remains the predominant method for hypoxia detection in clinical settings.

Tissue sectioning provides clear and precise microscopic visualization of tumor tissues, offering distinct diagnostic advantages. However, conventional hypoxia-sensitive fluorescent probes typically require antibody-based post-staining procedures following sectioning. Moreover, time-consuming staining procedures expose tissue sections to oxygen-rich ambient air, often altering the hypoxia levels from those present at the time of sampling 34. Given that hypoxia is highly sensitive to environmental changes, the accuracy of traditional staining-based hypoxia assessments remains suboptimal. In contrast, *in vivo* hypoxia imaging is certainly considered as the ideal approach due to its real-time capability. However, whole-body imaging in live animals only a macroscopic view of tumor presence and lacks the resolution to precisely localize hypoxic regions 35. In the face of these challenges, we wondered whether we could develop a new modality of hypoxia detection by taking advantage of the strengths and offsetting the weaknesses of *in vivo* detection strategy and tissue sectioning technique. We proposed to develop a nanoprobe to enable instant and stain-free detection of hypoxic niches in tumor sections. Following intravenous administration, the nanoprobe with tumor hypoxia-responsive luminescence characteristics is expected to be efficiently accumulated in tumors. Subsequently, tumor sections would be obtained for immediate fluorescence imaging without any staining and incubation processing, enabling point-of-care hypoxia detection.

**Figure 1:**
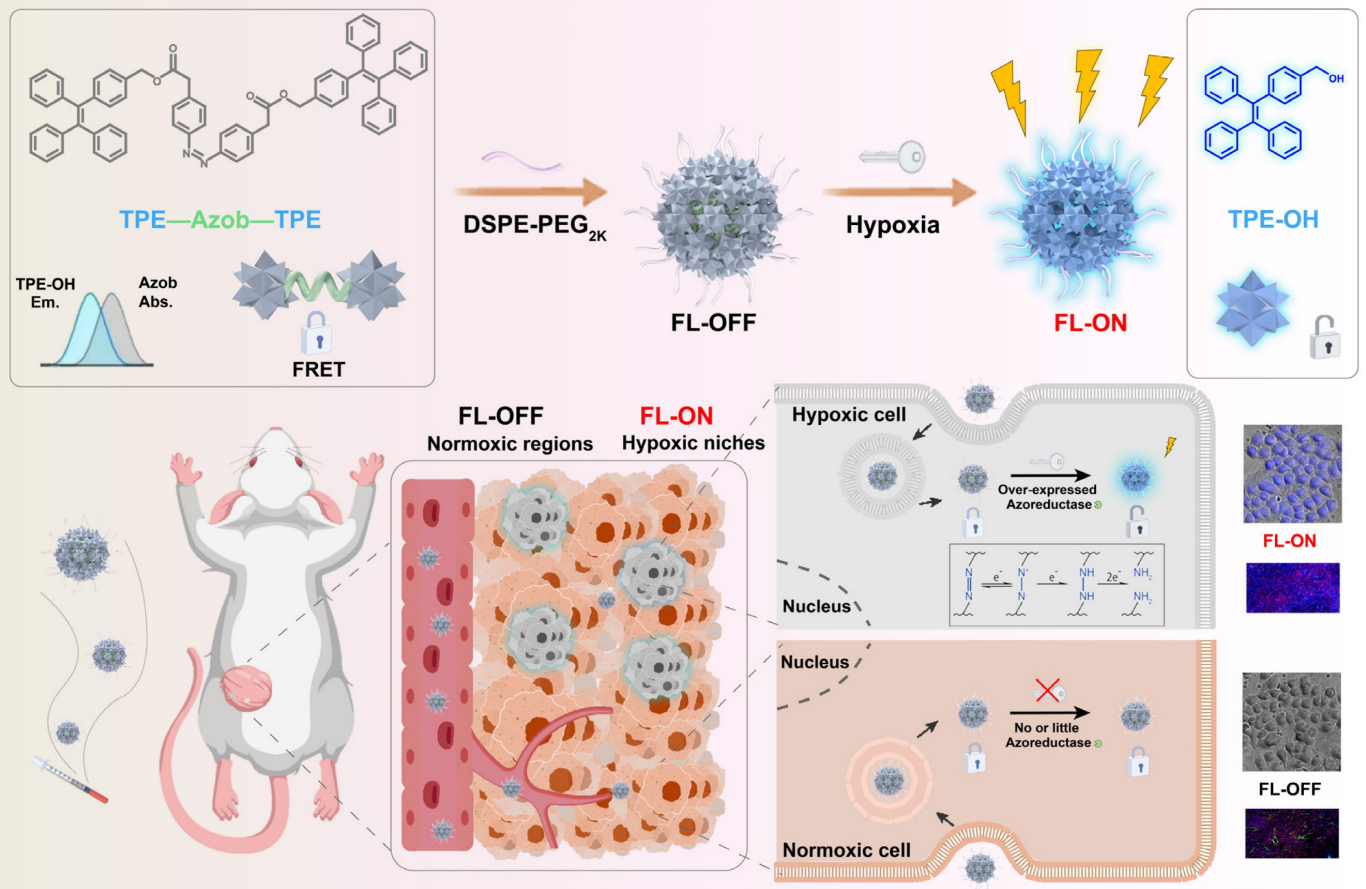
Schematic representation of a FRET-based off-on AIE nanoprobe (p-TNNT NAs) enables instant and stain-free detection of hypoxic niches in tumor sections.

To test our hypothesis, we elaborately designed and synthesized an instantly hypoxia-detecting nanoprobe (p-TNNT NAs) based on the combined principles of AIE, Förster resonance energy transfer (FRET) effects and hypoxia-sensitive azobenzene (Azo) bond (Figure 1). To our knowledge, this is the first attempt to construct a carrier-free AIE nanoprobe by integrating AIE fluorescence imaging, azobenzene prodrug strategy, small-molecule nanoassembly technique, and FRET effect into one nanosystem. Such a stain-free monitoring modality drives a conceptual step forward in fast and accurate tumor hypoxia detection. In detail, we constructed an azobenzene-bridged tetraphenylethene (TPE) dimer (TNNT) that could self-assemble into stable nanoassemblies (NAs) in aqueous environments. TPE was selected as the fluorophore due to its ability to engage in FRET interactions with the Azo linkage, which served as a hypoxia-sensitive quencher in the dimer (TNNT, Figure 1). Although TPE with short-wavelength fluorescence emission is not suitable for *in vivo* imaging, its fluorescence characteristics are well-suited for tissue section imaging using conventional fluorescence detection instruments. Given the inability of whole-animal imaging to localize hypoxic niches precisely, we aimed to developed a FRET-based off-on AIE nanoprobe for instant and stain-free detection of hypoxic niches in tumor sections after intravenous administration. Notably, the abundant presence of aromatic groups in TPE and Azo endowed TNNT with excellent nanoassembly capability. Based on the nanoassembly capacity of TNNT, we then fabricated stable dimeric NAs (p-TNNT NAs) using DSPE-PEG_2K_ as the surface modifier. The AIE nanoprobe (p-TNNT NAs) was found to efficiently accumulate in tumors after intravenous injection. We found that the FRET effect between TPE and Azo "turned off" the AIE luminescence of p-TNNT NAs under normoxic conditions. The fluorescence could be readily "turned on" following Azo cleavage under hypoxic conditions. Finally, such a uniquely engineered off-on AIE nanoprobe was used to detect hypoxic niches in the tumor sections of 4T1 breast tumor-bearing mice. Compared to pimonidazole hydrochloride (HP3, a conventional hypoxia probe), p-TNNT NAs exhibited markedly stronger fluorescence signals in tumor sections. Moreover, this nanoprobe was able to pinpoint the changes in hypoxia in tumors of varying sizes (from 40 to 200 mm³). Immunofluorescence staining of CD31 further validated that fluorescence mainly occurred in regions distant from blood vessels. Importantly, p-TNNT NAs allowed for instant and stain-free hypoxia detection upon sectioning, eliminating the need for time-consuming staining procedures. This strategy would undoubtedly improve the accuracy and efficiency of hypoxia detection in pathological analysis.

## Results and discussion

### Design and synthesis of a FRET-based AIE dimer

**Figure 2:**
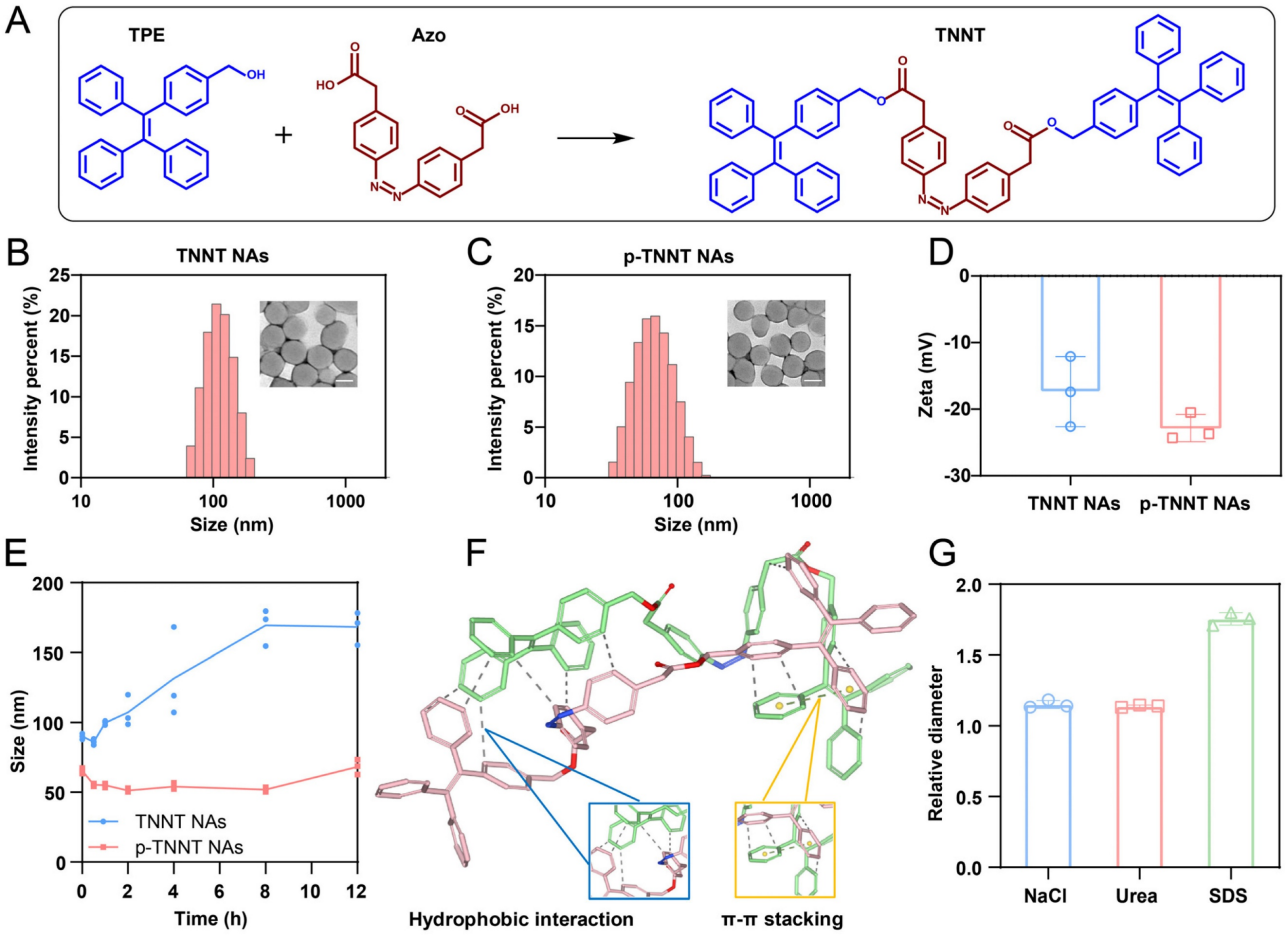
Characterization of TNNT NAs and p-TNNT NAs. (A) Structural formula of TPE, Azo and TNNT, as well as schematic representation of the TNNT synthesis process. (B-C) Size distribution profiles and TEM photos of TNNT NAs and p-TNNT NAs. Scale bar: 100 nm. (D) Zeta potential of TNNT NAs and p-TNNT NAs (n = 3). (E) Colloidal stability in PBS (pH 7.4) with 10% FBS of TNNT NAs and p-TNNT NAs (n = 3). (F) Molecular docking simulations results illustrating the molecular interactions involved in the self-assembly of TNNT. (G) The particle size changes of TNNT NAs exposed to NaCl (10 mM), urea (10 mM) and SDS (10 mM), respectively (n = 3).

In this project, we aimed to design and synthesize a hypoxia-activatable AIE probe featuring off-on fluorescence switching and self-assembly capability. Azo, a classic hypoxia-responsive linker, has been extensively employed in hypoxia-sensitive probes or prodrugs due to its unique ability to undergo bioreduction under low-oxygen conditions, leading to cleavage of the Azo bond 36-38. The selection of an appropriate AIEgen was critical to ensure efficient fluorescence quenching via FRET effect between AIEgen and Azo. To achieve effective FRET-mediated quenching, TPE with fluorescence spectrum overlap with the absorption spectrum of Azo was chosen as the AIEgen moiety (Figure S1). Additionally, while TPE was not suitable for *in vivo* imaging due to its short fluorescence emission wavelength, its fluorescence characteristics were well-suited for tissue section imaging. Tissue section imaging typically employed various types of fluorescence microscopes, with blue, green, and red channels being the most commonly used. TPE emitted fluorescence at 460 nm, which could be readily detected through the blue channel. Based on these considerations, TPE and Azo were covalently conjugated via esterification (Figure 2A and Figure S2), yielding a dimeric AIE probe (TNNT) in response to hypoxic tumor microenvironment. Successful synthesis of TNNT was validated through ^1^H NMR and ^13^C NMR analysis (Figure S3 and Figure S4).

### Molecular engineering of an AIE nanoprobe

Interestingly, we observed that TNNT could self-assemble into spherical nanoparticles with an average diameter of approximately 110 nm (Figure 2B and Table S1). To enhance stability and facilitate *in vivo* delivery efficiency, DSPE-PEG_2K_ was modified onto the surface of TNNT NAs. DSPE-PEG_2K_ has been widely employed as a surface modifier and stabilizer for small-molecule prodrug NAs 39. Notably, the PEGylated NAs (p-TNNT NAs) exhibited smaller particle sizes and greater electronegativity compared to the non-PEGylated TNNT NAs (Figure 2C-D). This size reduction of p-TNNT NAs should be attributed to the formation of a more compact nanoassembly with the help of amphiphilic PEG polymer (DSPE-PEG_2K_) 40, 41. In order to screen out the optimal ratio of DSPE-PEG_2K_, varying amounts of DSPE-PEG_2K_ were used in the preparation of PEGylated TNNT NAs. After a comprehensive evaluation of particle diameter, polydispersity index (PDI), and colloidal stability, we found that 20 wt% DSPE-PEG_2K_ was optimal for fabricating the PEGylated TNNT NAs, designated as p-TNNT NAs (Figure S5 and Table S1). Subsequently, we evaluated the stability of the NAs under simulated physiological conditions. As shown in Figure 2E and S6A, p-TNNT NAs maintained a consistent particle size profile for up to 12 h in PBS (pH 7.4), PBS (pH 7.4) containing 10% FBS, PBS (pH 7.4) with 10% Sprague Dawley rat plasma. To better mimic the *in vivo* environment, we explored the stability of p-TNNT NAs in PBS (pH 7.4) containing Sprague Dawley rat plasma. As shown in Figure S6B, p-TNNT NAs displayed good stability in PBS (pH 7.4) containing 10% Sprague Dawley rat plasma. Good stability certainly benefits the *in vivo* delivery and tumor-specific accumulation of the nanoprobe. In contrast, the unmodified TNNT NAs displayed significant instability. To evaluate long-term stability, we monitored the particle size changes of TNNT NAs and p-TNNT NAs stored at 4℃ for seven days. As shown in Figure S7, p-TNNT NAs exhibited negligible changes in particle size, indicating excellent long-term colloidal stability. By contrast, TNNT NAs without PEGylation modification showed poor storage stability.

We next investigated the self-assembly mechanism of TNNT. Computational docking simulations revealed that hydrophobic interactions and π-π stacking interactions were the dominant intermolecular forces driving the nanoassembly of TNNT (Figure 2F). To further validate these interactions, sodium dodecyl sulfate (SDS), sodium chloride (NaCl) and urea were employed to disassemble the NAs by disrupting hydrophobic interaction, electrostatic force and hydrogen bonding, respectively. The NAs exhibited remarkable stability upon incubation with NaCl and urea, as evidenced by negligible changes in particle size. This observation suggested that electrostatic interactions and hydrogen bonding played a negligible role in the self-assembly process of TNNT. In contrast, incubation with SDS resulted in a pronounced increase in particle size. The resulting alterations in particle size conclusively affirmed the pivotal contribution of hydrophobic interactions in driving nanoassembly of TNNT (Figure 2G).

### *In vitro* off-on AIE luminescence

**Figure 3:**
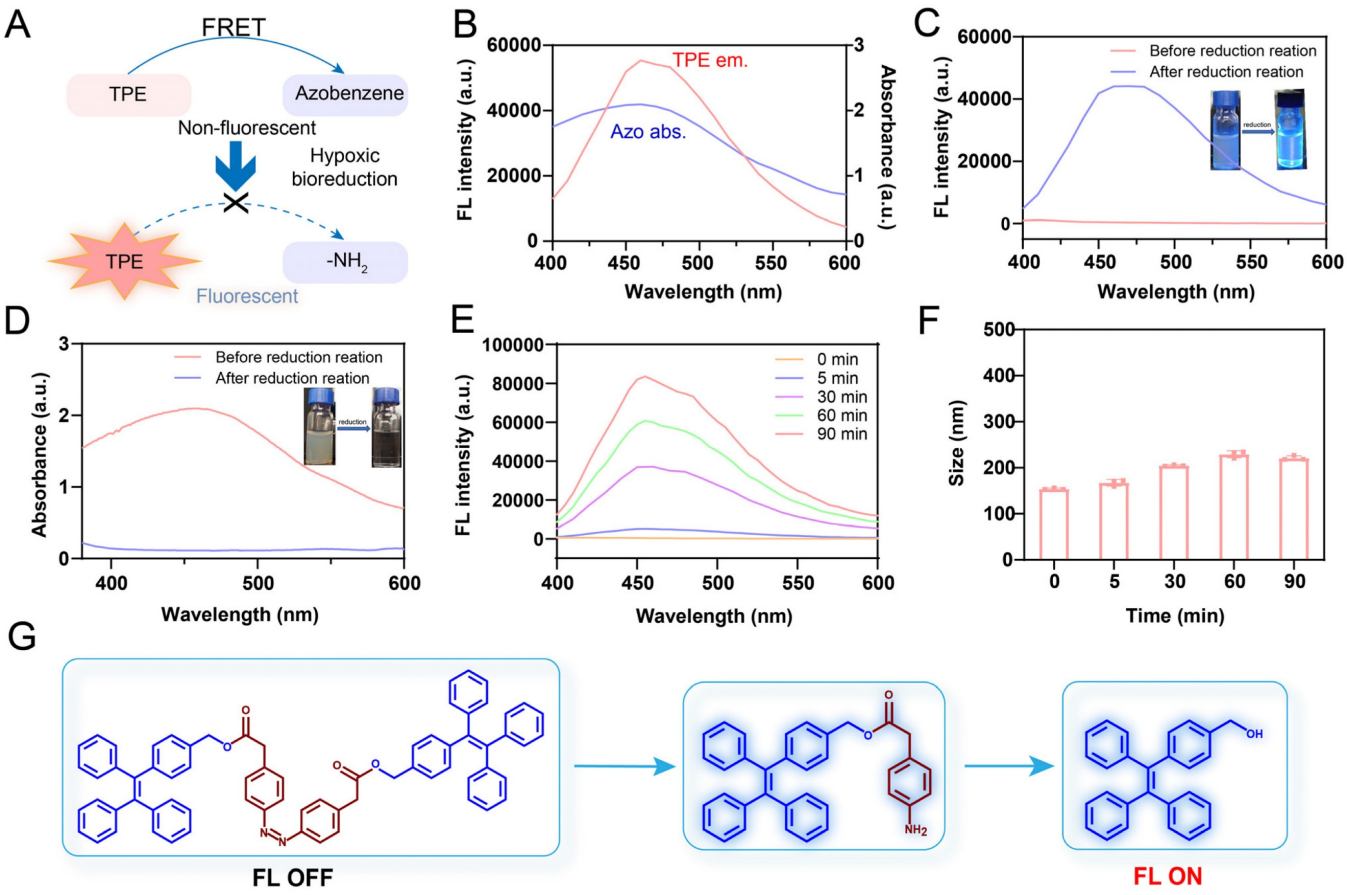
Characterization of the fluorescence properties of p-TNNT NAs *in vitro*. (A) Schematic representation of “off-on” fluorescence of TPE in p-TNNT NAs. (B) Fluorescence emission spectrum of TPE and absorption spectrum of Azo. (C-D) UV-vis absorption spectrum, fluorescence emission spectrum and appearance photos under daylight and UV light of p-TNNT NAs before and after reduction reaction (p-TNNT NAs: 10 μM). (E) Fluorescence recovery of nanoprobe (p-TNNT NAs: 10 μM) upon incubation with Na_2_S_2_O_4_ (20 mM). (F) The particle size of nanoprobe (p-TNNT NAs: 10 μM) upon incubation with Na_2_S_2_O_4_ (20 mM) (n = 3). (G) Schematic diagram of bond fracture under hypoxic conditions.

We first assessed the AIE luminescence behavior of TPE in a series of THF/H_2_O mixtures. In pure THF, TPE exhibited negligible fluorescence emission, due to non-radiative energy loss resulting from unrestricted intramolecular motion in the solution. However, as the water content increased beyond 70%, the fluorescence intensity gradually increased, suggesting that the restriction of molecular motion activated the AIE effect (Figure S8). Given the strong spectral overlap between the absorption spectrum of Azo and the emission spectrum of TPE, Azo was expected to serve as an efficient quencher of TPE fluorescence. Furthermore, the covalent conjugation between Azo and TPE shortened the intermolecular distance, thereby facilitating FRET-mediated quenching in the TNNT construct (Figure 3A-B) 42. Owing to the FRET effect, TNNT remained in a fluorescence-quenched state even after forming nanoaggregates. Next, we validated the "off-on" fluorescence behavior of p-TNNT NAs. Under excitation at 365 nm, the fluorescence spectrum of p-TNNT NAs exhibited no detectable emission confirming that Azo moiety efficiently quenched TPE fluorescence. Upon incubation with sodium dithionite (Na_2_S_2_O_4_, a commonly used agent to simulate hypoxic conditions), the fluorescence was restored, showing a peak at 460 nm (Figure 3C). This fluorescence recovery could be ascribed to Azo cleavage induced by Na_2_S_2_O_4_, which disrupted the FRET effect between TPE and Azo. As shown in Figure 3D, the absorption peak of Azo disappeared after Na_2_S_2_O_4_ treatment, further validating the cleavage of Azo. Mass spectrometry and FTIR further identified the reduction products as TPE-NH_2_ and TPE in the presence of Na_2_S_2_O_4_ (Figure 3G and S9-S11). Subsequently, we conducted a fluorescence response specificity assay. As shown in Figure S12, the p-TNNT NAs underwent fluorescence recovery only under hypoxic conditions, while there was almost no fluorescence change observed under other conditions. These results confirmed that p-TNNT NAs possess hypoxia-specific “off-on” fluorescence behavior. The key factors affecting the fluorescence intensity were the concentration of p-TNNT NAs and the degree of the simulated hypoxic environment. Therefore, we investigated the relationship between p-TNNT NAs concentration and fluorescence intensity. As shown in Figure S13, as the p-TNNT NAs concentration increases, the fluorescence intensity after activation gradually increases. Additionally, we further investigated the relationship between hypoxia levels and fluorescent intensity of p-TNNT NAs. As shown in Figure 3E and S14, the fluorescence intensity of p-TNNT NAs increased in a Na_2_S_2_O_4_ concentration- and time-dependent manner, indicating that the fluorescence recovery rate is closely correlated with p-TNNT NAs concentration, hypoxia levels and exposure duration. Notably, the NAs maintained colloidal stability for up to 90 min during co-incubation, preserving the aggregation state of AIEgen and ensuring stable nanoassembly fluorescence (Figure 3F).

### *In vitro* hypoxia detection in tumor cells and MCTS

**Figure 4:**
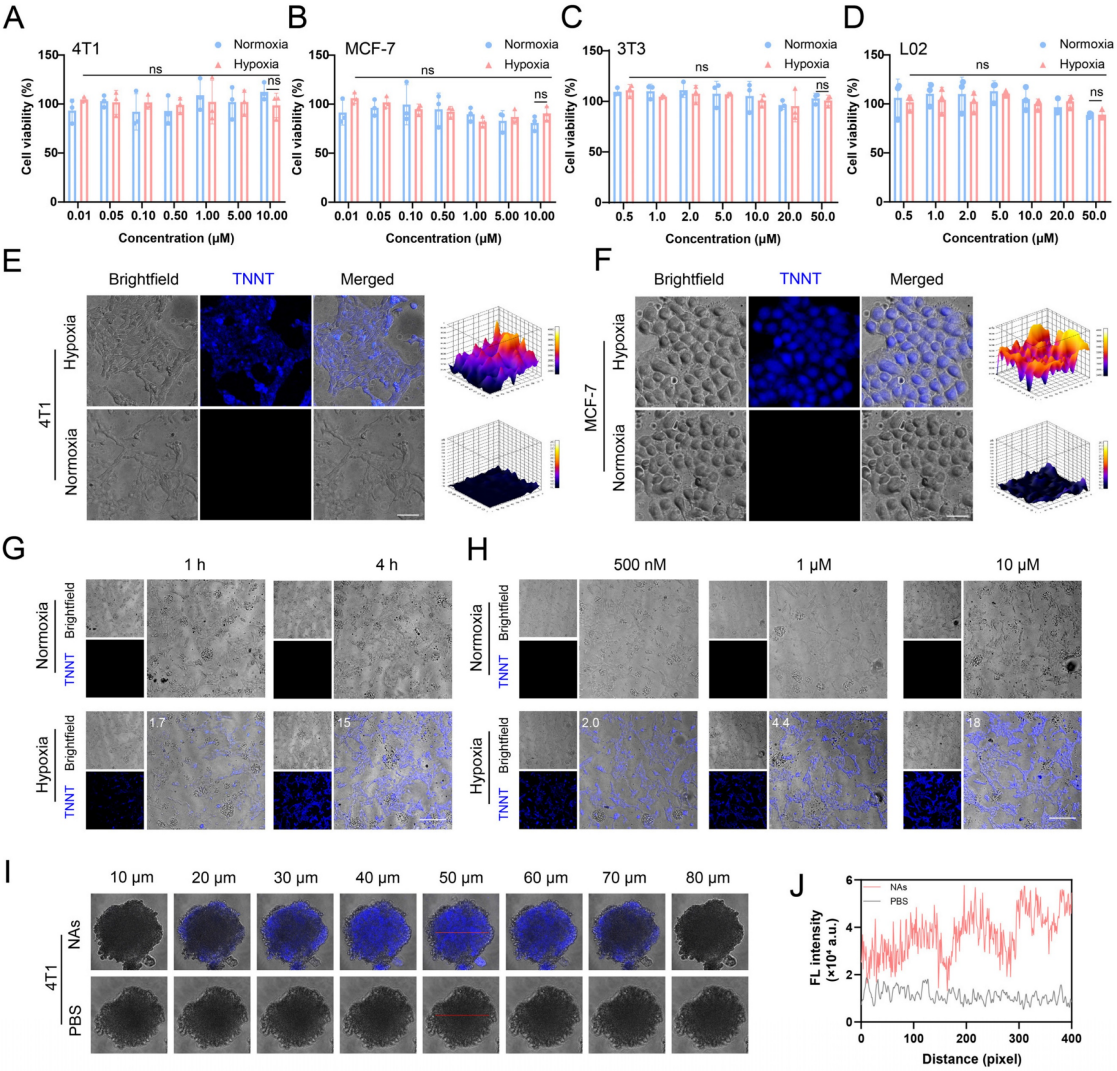
Characterization of the fluorescence properties of p-TNNT NAs at the cellular level. (A-D) Cytotoxicity of p-TNNT NAs in 4T1, MCF-7, 3T3 and L02 cells under normoxia or hypoxia (n = 3). (E-F) The CLSM images of p-TNNT NAs in 4T1 and MCF-7 cells under hypoxia or normoxia (p-TNNT NAs: 10 μM). Scale bar: 50 μm. (G) The CLSM images and fluorescence semiquantitative analysis of p-TNNT NAs in 4T1 cells under hypoxia or normoxia at different time points (p-TNNT NAs: 10 μM). Scale bar: 200 μm. (H) The CLSM images and fluorescence semiquantitative analysis of p-TNNT NAs in 4T1 cells under hypoxia or normoxia at different concentrations of p-TNNT NAs. Scale bar: 200 μm. (I) The CLSM images of p-TNNT NAs and PBS in 4T1 multicellular tumor spheroids (p-TNNT NAs: 10 μM). Scale bar: 50 μm. (J) Fluorescence quantitative analysis of images at a depth of 50 microns in I (red line).

Building on the favorable self-assembly capacity, colloidal stability and hypoxia-responsive fluorescence properties of p-TNNT NAs, we further evaluated their hypoxia-responsive fluorescence recovery in tumor cells. Ideal nanoprobes must exhibit minimal toxicity to ensure safety for biomedical applications. Accordingly, we first assessed the cytotoxicity of p-TNNT NAs in 4T1 and MCF-7 cancer cells, as well as in normal 3T3 fibroblasts and L02 hepatocytes under both normoxic and hypoxic conditions. As shown in Figure 4A-D, p-TNNT NAs exhibited negligible cytotoxicity across all tested cell lines under both normoxic and hypoxic conditions. Good biocompatibility of p-TNNT NAs ensures their safety for functioning as a potential fluorescence probe for hypoxia detection.

Next, we investigated the hypoxia-responsive fluorescence behavior of p-TNNT NAs in tumor cells. Confocal microscopy images of 4T1 and MCF-7 cells treated with p-TNNT NAs under normoxic and hypoxic conditions were shown in Figure 4E-F. Under normoxic conditions (20% O_2_), negligible fluorescence was detected in both 4T1 and MCF-7 cells, which could be attributed to the minimal expression of azoreductase in normoxic cells. In contrast, under hypoxic conditions (1% O_2_), these two kinds of tumor cells displayed a significant enhancement of fluorescence signals. This fluorescence activation was due to the upregulation of azoreductase in the hypoxic tumor microenvironment, which cleaved the Azo bonds, disrupted the FRET effect and restored the fluorescence of TPE 43, 44. In addition, we further evaluated the fluorescence behavior of p-TNNT NAs in 4T1 cells under varying incubation times and concentrations, both in hypoxic and normoxic conditions. As shown in Figure 4G, fluorescence intensity gradually increased with prolonged incubation time under hypoxic conditions, indicating a time-dependent fluorescence recovery of the probe. This phenomenon could be attributed to the gradual cleavage of Azo bonds by intracellular azoreductase, thus disrupting the FRET effect and restoring the fluorescence of TPE. Moreover, a concentration-dependent fluorescence enhancement was observed (Figure 4H), with 4T1 cells treated with increasing concentrations of p-TNNT NAs (500 nM to 10 μM) exhibiting progressively stronger intracellular fluorescence. To further validate these observations, we performed semiquantitative analysis of the confocal fluorescence images in Figure 4G and 4H using ImageJ, and the quantified results were included in the corresponding figures. These results demonstrated that the fluorescence restoration of p-TNNT NAs showed dynamic changes in a time- and dose-dependent manner under hypoxic conditions. To further assess the performance of p-TNNT NAs in a more physiologically relevant setting, we established a multicellular tumor spheroid (MCTS) model. MCTS, a 3D cell culture system, better mimics* in vivo* tumor growth, including the formation of hypoxic and apoptotic/necrotic regions due to oxygen and nutrient gradients. As shown in Figure 4I-J, significantly higher fluorescence was observed in MCTS treated with p-TNNT NAs compared to the control group. In addition, we randomly selected two regions at a depth of 50 μm (indicated by red lines in Figure S15A-B) and performed semi-quantitative analysis using Image J. As shown in Figure S15D-E, fluorescence intensity was lower at the spheroid periphery but markedly higher in the core. Furthermore, we delineated the boundary between the outer and inner regions with a white dashed line (Figure S15C) and analyzed fluorescence along this interface. The results showed a gradual increase in intensity from the periphery to the center (Figure S15F), confirming that the AIE probe was specifically activated in hypoxic regions. These findings demonstrated that p-TNNT NAs could act as an off-on fluorescence sensor for detecting hypoxic conditions in 3D tumor cultures, and was expected to provide a promising tool for studying tumor hypoxia in more complex *in vitro* models.

### *In vivo* pharmacokinetics and biodistribution after intravenous injection

As previously discussed, *in vivo* real-time imaging provided only broad tumor visualization without precise localization of hypoxic regions. Although* ex-vivo* tissue sectioning could provide a more accurate representation of the degree and distribution of hypoxia, it was often compromised by alterations in hypoxia levels during prolonged exposure to oxygen-rich environments throughout post-processing steps, such as staining and washing. For instance, when using commercially available hypoxia probes, the post-processing protocol typically required over 15 h of sequential treatments even after frozen sections. As detailed in the HP3 workflow, critical steps including overnight primary antibody incubation (12-16 h at 4°C), secondary antibody conjugation (1-2 h), and repeated PBS washes (6-8 cycles, 5 min each) collectively extend the exposure of tissue sections to ambient oxygen. Such prolonged oxidative exposured risks altering the original hypoxia-driven pimonidazole-protein adducts, particularly in marginally hypoxic regions. 45-47.

**Figure 5:**
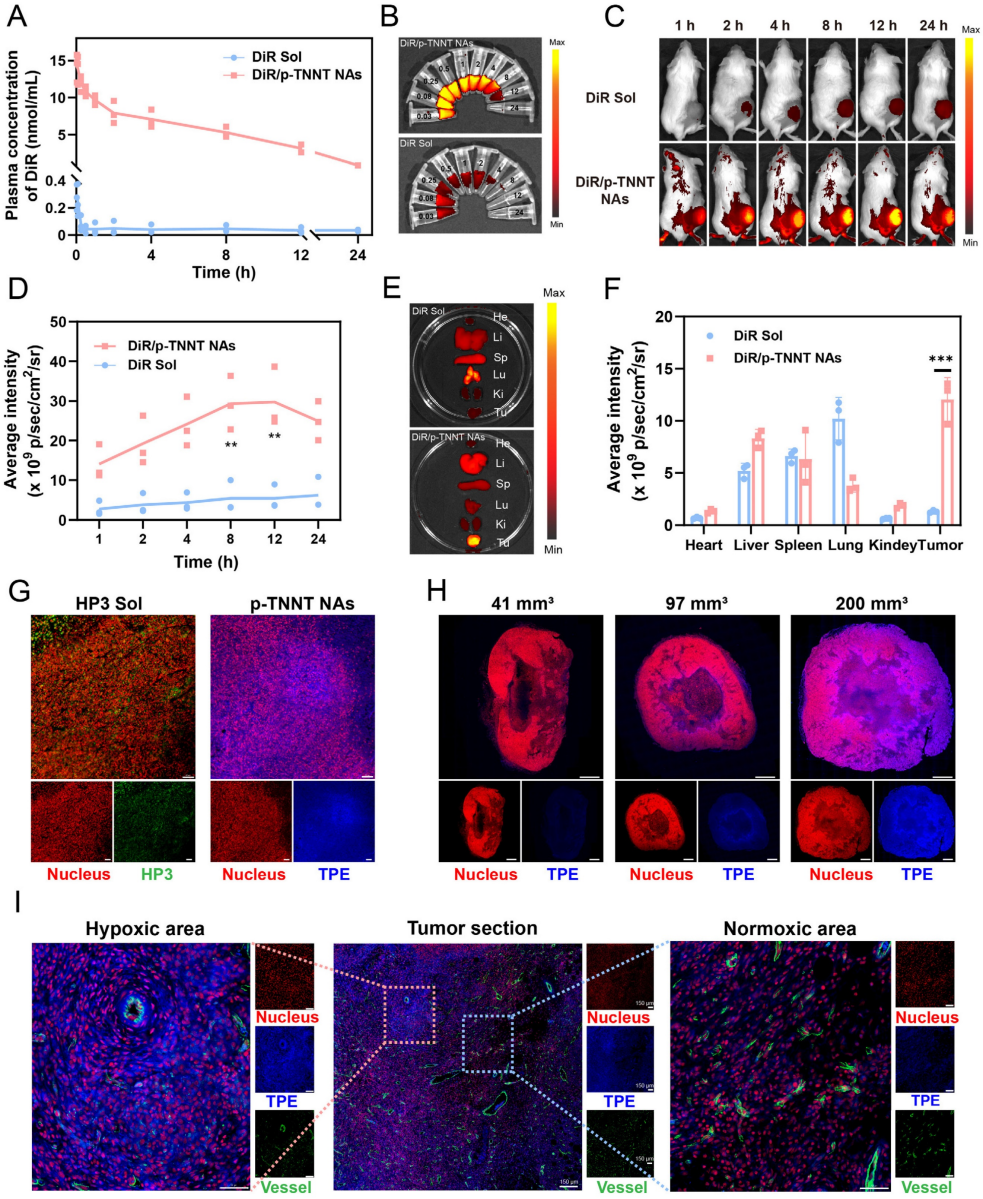
Pharmacokinetics, biodistribution and instant and stain-free detection of hypoxic niches in tumor sections. (A) Plasma concentration-time profiles of DiR/p-TNNT NAs and DiR Sol post a single intravenous administration (n = 3). (B) Images of pharmacokinetic plasma of DiR/p-TNNT NAs and DiR Sol. (C) *In vivo* NIR imaging of 4T1-bearing BALB/c mice administered DiR Sol or DiR/p-TNNT NAs (n = 3). (D) Quantification of the average fluorescence intensity of C. (E) *Ex vivo* NIR imaging of key tissues and tumors following 12 h treatment of DiR/p-TNNT NAs and DiR Sol (n = 3). (F) Quantification of tumor fluorescence signals in E. (G) The immunofluorescent staining of tumor slices after treated with HP3 Sol or p-TNNT NAs. Scale bar: 50 µm. (H) The immunofluorescent staining of tumor slices in different sizes after treated with p-TNNT NAs. Scale bar: 1 mm. (I) The immunofluorescent staining of tumor slices after treated with p-TNNT NAs (CD31 to stain blood vessels, PI to stain nuclei, and TPE to characterize hypoxic sites). Scale bar: 50 µm. Statistical significance was calculated by t-test: ** P < 0.01, *** P < 0.001.

To overcome these limitations, we proposed a stain-free strategy for hypoxia detection in *ex-vivo* tumor sections. A FRET-shielded AIE nanoprobe was administered intravenously and specifically activated under hypoxic conditions. Tumor sections were excised at the time of peak probe accumulation and immediately subjected to fluorescence imaging using standard detection equipment, eliminating the need for post-processing steps such as staining or antibody incubation. This strategy provided a new approach for hypoxia detection, ensuring both accuracy and convenience. As it was widely known that PEGylated nanosystems offer a distinct advantage in long systemic circulation time and high tumor accumulation. We expected that the PEGylated AIE off-on nanoprobe (p-TNNT NAs) would possess favorable pharmacokinetics properties and efficient tumor accumulation, which was essential for satisfactory tumor hypoxia detection. To evaluate the pharmacokinetic behavior of the probe, we utilized a DiR-labeled version (DiR/p-TNNT NAs), as the fluorescence of native p-TNNT NAs was quenched by Azo-mediated FRET effect 48. DiR/p-TNNT NAs were systemically administered via tail vein injection into Sprague Dawley rats, and the pharmacokinetic profiles were assessed by measuring DiR concentrations in plasma at different time points. As shown in Figure 5A-B and Table S2, DiR/p-TNNT NAs significantly prolonged the circulation time of DiR in the blood compared to free DiR solution (DiR Sol), potentially arising from optimized colloid stability and PEGylation modification.

Subsequently, we explored the biodistribution of DiR/p-TNNT NAs in 4T1 breast tumor-bearing BALB/c mice by *in vivo* imaging system (IVIS). Although the AIE fluorescence of p-TNNT NAs could be selectively activated in hypoxic tumor regions, it was unsuitable for evaluating the distribution of nanoprobes in normal tissues. Therefore, DiR-labeled nanoprobe (DiR/p-TNNT NAs) were employed to track nanoprobe biodistribution. As shown in Figure 5C-F, DiR/P-TNNT NAs exhibited significantly stronger fluorescence signals in tumor sites compared to DiR Sol, demonstrating excellent tumor-specific accumulation of the PEGylated NAs. The *Ex-vivo* distribution data showed that DiR/p-TNNT NAs exhibited the strongest fluorescence intensity at 12 h post-injection (Figure 5E-F). Based on this result, in subsequent experiments, we obtained tumor sections at 12 h post-injection for hypoxia detection. Taken together, these *in vivo* results suggested that the nanoprobe performed high delivery efficiency *in vivo*, laying a solid foundation for the development of stain-free nanoprobes for instant tumor hypoxia detection.

### Instant and stain-free detection of hypoxic niches in tumor sections

The satisfying nanoassembly, colloidal stability, hypoxia-responsive off-on AIE fluorescence characteristics *in vitro*, pharmacokinetics and biodistribution *in vivo* encouraged us to further evaluate its hypoxia detection capability in tumor sections. In this section, we first validated the ability of p-TNNT NAs to instantly detect tumor hypoxia by observing the fluorescence signals of tumor tissue sections after intravenous injection to 4T1 breast tumor-bearing mice. Furthermore, we selected HIF-1α as a well-established molecular marker to identify hypoxic niches. We then compared its distribution with that of the probe-labeled areas to further validate the *in vivo* efficacy of the probe. The results demonstrated a high degree of spatial overlap, confirming the probe's ability to accurately detect tumor hypoxia (Figure S16). A commonly used hypoxia detection probe (HP3) was used as the control. Tumor tissues were harvested and immediately cryosectioned for fluorescence imaging analysis after intravenous administration of p-TNNT NAs or HP3 solution at equimolar doses of TPE and HP3, respectively. As shown in Figure 5G and Figure S17, p-TNNT NAs exhibited significantly higher fluorescence intensity than that of HP3 solution under the same conditions, which should be ascribed to the long circulation time, high tumor accumulation, and tumor-specific off-on fluorescence characteristics of p-TNNT NAs. These results confirmed the feasibility of a stain-free AIE nanoprobe for detecting hypoxia niches in tumor sections.

Increasing evidence showed that tumor hypoxia was closely related to tumor size and disease progression. Small tumors usually had low hypoxic levels, whereas large tumors have hypoxic niches 49, 50. We were curious whether this probe (p-TNNT NAs) could distinguish the levels of hypoxia in tumors of different sizes. To further assess the sensitivity of p-TNNT NAs in detecting tumor hypoxia in small and large tumors, we selected tumors of different volumes (approximately 40 mm^3^, 100 mm^3^ and 200 mm^3^) for section analysis (Table S3). As shown in Figure 5H and Figure S18, p-TNNT NAs exhibited hypoxia detection capability in tumors spanning a size range of 40 - 200 mm³. Subsequently, we performed CD31 staining to observe the vascular distribution. As shown in Figure 5I, it turned out that the blue fluorescence of p-TNNT NAs was mainly located in tumor hypoxic regions away from the blood vessels (green fluorescence). In addition, we further investigated the safety of the p-TNNT NAs. As shown in Figures S19-20, no hemolysis was observed in the p-TNNT NAs. Moreover, hepatorenal function parameters and histological analysis of major organs (heart, liver, spleen, lung, and kidney) revealed no significant damage or abnormalities (Figure S21-22), further confirming the biocompatibility of the p-TNNT NAs. In conclusion, these findings provided solid evidence that such a stain-free AIE nanoprobe was able to instantly detect hypoxic niches in tumor sections following intravenous injection, which had the potential to provide instant and useful data for diagnosis, as well as for the rational design and optimization of treatment regimens in clinical cancer treatment.

## Conclusions

In short, we developed self-assembled stain-free nanoprobe (p-TNNT NAs) that integrated the advantages of *in vivo* imaging and tissue sectioning for instant and precise detection of hypoxic regions within tumor sections. Both *in vitro* and *in vivo* studies verified the feasibility of p-TNNT NAs as a stain-free hypoxia probe. *In vitro*, p-TNNT NAs exhibited excellent colloidal stability and hypoxia-responsive fluorescence activation. *In vivo*, the nanoprobe exhibited prolonged blood circulation time and efficient tumor accumulation, laying the foundation for instant and precise detection of hypoxic niches in tumor sections. One of the key strengths of p-TNNT NAs was their ability to combine the advantages of *in vivo* delivery and *ex-vivo* section imaging techniques, enabling antibody-free fluorescence imaging and eliminating the need for post-processing staining. As a result, p-TNNT NAs could be used to instantly detect tumor hypoxia, completely preventing interference from external circumstances during section staining, thereby enabling more accurate detection. Collectively, these findings underscore the clinical potential of p-TNNT NAs as a practical tool for hypoxia detection in tumor sections, offering insights for developing precise and rapid detection strategies for tumor hypoxia.

## Methods

### Materials

TPE was bought from Shanghai Bidepharmatech Co., Ltd. Azo was bought from Zhengzhou Acme Chemical Co., Ltd. EDCI, DMAP, HOBT was bought from Energy Chemical Co., Ltd. Hypoxyprobe™ Omni Kit was supplied by Hypoxyprobe, Inc. Na_2_S_2_O_4_ was purchased from Shanghai Macklin Co., Ltd. DiR, RPMI 1640 cell culture medium, penicillin-streptomycin, FBS and MTT were obtained from Dalian Meilun Biotechnology Co., Ltd. DSPE-PEG_2K_ was supplied by AVT Shanghai Pharmaceutical Technology Co., Ltd. Cell culture dishes and plates were purchased from Wuxi NEST Biotechnology Co., Ltd. Other solvents and chemicals applied in the present study were of analytical reagent grade.

### Cell culture

Human breast carcinoma cells (MCF-7), mouse breast carcinoma cells (4T1), mouse fibroblast cells (3T3) and human liver cells (L02) were cultured in 1640, DMEM, DMEM/F12 and 1640 respectively, all supplemented with penicillin (100 units mL^-1^), streptomycin (100 μg mL^-1^) and 10% fetal bovine serum (FBS) under a humidified atmosphere of 5% CO_2_ at 37 °C.

### Synthesis of TNNT

Azo (29.8 mg), HOBT (27.0 mg), and EDCI (57.5 mg) were dispersed in dimethyl formamide (10 mL) and dichloromethane (10 mL). In ice-bath conditions, the mixture was stirred continuously for 2 h. Then, DMAP (6.1 mg) and TPE (108.7 mg) were supplemented to the reaction mixture and maintained for one day under nitrogen under 25 ℃. The extent of reaction coupled with real-time TLC tracking. Preparative liquid chromatography (100% acetonitrile for mobile phase) was utilized to make pure TNNT, gaining an orange solid (productivity 34.7%).

### Preparation and characterization of TNNT NAs

The non-PEGylated NAs (TNNT NAs) was fabricated by single-step nanoprecipitation method. To put it simply, TNNT (1.0 mg) was dispersed in a 200 μL of ethanol/tetrahydrofuran mixture (1:1, v/v). Next, the TNNT solution was added drop by drop in 2 mL purified water under constant robust stirring about 3 min. After that, the ethanol/tetrahydrofuran mixture in the nanosystems was evaporated using a rotary evaporator. Finally, these prepared NAs were stored in the refrigerator at 4°C. To determine the optimal proportion of DSPE-PEG_2K_ for nanoparticle PEGylation, TNNT NAs were prepared with varying weight percentages of DSPE-PEG_2K_ (10%, 20%, 30%, w/w). The resulting PEGylated nanoparticles were characterized for their hydrodynamic diameter and PDI using dynamic light scattering (DLS). Additionally, their stability was monitored over time in PBS (pH 7.4) and PBS (pH 7.4) with 10% FBS at 37 °C to assess PEGylation efficiency. Based on the comparative analysis, the formulation containing 20 wt% DSPE-PEG_2K_ exhibited the most favorable physicochemical properties and was selected as the optimized formulation, designated as p-TNNT NAs.

The Zetasizer was applied to evaluate Zeta potential and particle size of both TNNT NAs and p-TNNT NAs. The structure of both TNNT NAs and p-TNNT NAs was examined utilizing transmission electron microscopy after staining with 2% phosphotungstic acid.

### Colloidal stability

In order to study the stability under simulated physiological conditions, variations in the size of TNNT NAs and p-TNNT NAs were monitored. Briefly, TNNT NAs (1 μmol/mL) and p-TNNT NAs (1 μmol/mL) remained in PBS (pH 7.4) with 10% FBS, incubated in a temperature-controlled orbital shaker. Their mean diameters after incubation were measured at the predesigned timepoints. For plasma stability assessment, p-TNNT NAs (1 μmol/mL) were incubated in PBS (pH 7.4) containing 10% freshly collected rat plasma at 37 °C. At predetermined time intervals, samples were withdrawn and analyzed by DLS to monitor potential changes in particle size. To evaluate the long-term storage stability, TNNT NAs and p-TNNT NAs were stored at 4 °C, and their particle sizes were recorded every day.

### Mechanisms of molecular self-assembly

The self-assembly mechanism of TNNT was investigated via molecular docking simulations conducted on the Yinfo Cloud Platform. Using the compound structure preparation utility, the 3D models of TNNT were initially generated and then optimized through energy minimization in the MMFF94 force field. Afterwards, small-molecule-small-molecule docking was carried out with the AutoDock Vina program. A semi-flexible docking approach was employed to obtain as many as 9 output conformations after an internal clustering process, allowing for a detailed analysis of the interactions. To further validate the presence of intermolecular interactions within the NAs, NaCl, SDS, and urea were applied to probe intermolecular forces. Following dispersion in NaCl, SDS, or urea solutions (10 mM each), TNNT NAs were incubated at 37 ℃ in an orbital shaker. Particle size dynamics were tracked over time using a Malvern ZetaSizer.

### UV/Vis and photoluminescence spectra

The UV/Vis and photoluminescence spectra of Azo Sol, TPE Sol and p-TNNT NAs were characterized by Varioskan Flash multimode microreader (BioTek, USA).

### FRET-switchable Off-on AIE luminescence

p-TNNT NAs (0.1、0.05、0.02、0.005 μg/mL) were dispersed to Na_2_S_2_O_4_ (0, 1, 2, 5, 10, 20 mM) and incubated at 37 ℃ for different times. The increase in fluorescence from TNNT was observed with a fluorescence spectrometer utilizing excitation at 320 nm and emission between 400 and 600 nm. Their particle size underwent periodic analysis via ZetaSizer measurements.

### Cytotoxicity assay

A density of 2×10³ 4T1, MCF-7, 3T3 and L02 cells per well were inoculated in 96-well plates, respectively. When the incubation lasted for 12 h in normoxic conditions, fresh media containing various concentrations of above formulations of p-TNNT NAs were utilized to replace old media. Then these cells were kept cultured for additional 48 h in either hypoxic or normoxic environment. Finally, thiazolyl blue tetrazolium bromide assay was applied to evaluate the cellular viability after treatments.

### *In vitro* hypoxia detection in tumor cells and multicellular tumor spheroids

A density of 2×10⁵ cells 4T1 and MCF-7 cells per dish were utilized in 35 mm dishes. Subsequent to 12 h of growth, cultured media was removed. Then the cells were maintained in fresh culture medium supplemented with p-TNNT NAs (10 μM) for 4 h in hypoxic or normoxic conditions. After discarding the drug-loaded media, adherent cells underwent triple washing with PBS (4 °C, pH 7.4) to eliminate residual compounds. Fluorescent labeling within the cytoplasm was then captured through confocal laser scanning microscopy.

4T1 cells (1×10^5^) were maintained in precoated 96-well agarose plates at 37℃ for 7 days to develop 3D tumor spheroids. Next, p-TNNT NAs (10 μM) were added to the precoated 96-well agarose plates and cultured for 12 h. Images were captured with confocal laser scanning microscopy.

### Animal studies

The use of animals is approved by the Animal Ethics Committee of Shenyang Pharmaceutical University (No. 19169).

### *In vivo* pharmacokinetics and biodistribution

We utilized Sprague-Dawley rats (180-220 g) to investigate pharmacokinetic characteristics of NAs (n = 3). The DiR/p-TNNT NAs were fabricated by single-step nanoprecipitation method. DiR (0.1 mg) TNNT (1.0 mg) DSPE-PEG_2K_ (20%, w/w) were dispersed in a 200 μL of ethanol/tetrahydrofuran mixture (1:1, v/v). Next, the solution was added drop by drop in 2 mL purified water under constant robust stirring about 3 min. After that, the ethanol/tetrahydrofuran mixture in the nanosystems was evaporated using a rotary evaporator. The rats received intravenous administration of DiR/p-TNNT NAs and DiR Sol, both normalized to a DiR-equivalent concentration of 1 mg·kg⁻¹. Blood samples (≈ 300 μL) were sequentially collected from rat subjects at predetermined intervals. Following immediate centrifugation (10,000 rpm, 3 min), plasma fractions were isolated and subjected to methanol-mediated protein precipitation for DiR extraction. Finally, plasma concentrations of DiR were established utilizing a multifunctional microplate reader with excitation at 748 nm and emission collected at 780 nm. The plasma separated at different time points was photographed under *in vivo* imaging system (Ex = 748 nm, Em = 780 nm).

A xenograft model of 4T1 breast cancer was established in female BALB/c mice to assess the *ex vivo* distribution of the NAs. In brief, 100 μL of PBS (pH 7.4) suspended with a total of 5×10^6^ cells were subcutaneously injected into the mice. DiR Sol and DiR/p-TNNT NAs were intravenously administrated to the mice at an equivalent DiR dose of 1 mg kg^-1^ until the tumor volume was about 400 mm^3^ (n = 3). At 12 h after dosing, the animals were euthanized, followed by collection of the heart, liver, spleen, lung, kidney and tumors. Finally, the fluorescent emissions from primary tissues and neoplastic lesions were characterized using* in vivo* imaging system (Ex = 748 nm, Em = 780 nm).

### Rapid detection of hypoxic niches in tumor sections after intravenous injection

Similarly, in the 4T1 breast cancer xenograft model, tumor volume was calculated using the formula: length × width × width / 2. Once the tumor volume reached 200 mm³, HP3 and p-TNNT NAs were administered systemically via tail vein injection. Tumors were excised 12 h later for sectioning and staining. Additionally, tumors of various sizes were selected, and p-TNNT NAs were delivered intravenously via tail vein injection. Tumors were excised 12 h post-injection for sectioning and staining.

### Safety evaluation

In the hemolysis assay, the red blood cell (RBC) suspensions were prepared by mixing 20 μL of RBCs with 1 mL of saline, and these suspensions were treated with p-TNNT NAs and TNNT NAs at equivalent concentrations and then incubated at 37°C (2 h). After centrifugation at 1800 × g for 5 min, the supernatants were collected and hemoglobin absorbance at 450 nm was measured using a microplate reader (n = 3).

Tumor-bearing mice were euthanized to evaluate *in vivo* biosafety. Orbital blood was collected for liver and kidney function tests. The five major organs (heart, liver, spleen, lungs, and kidneys) were collected. H&E staining was performed to examine tissue morphology and assess potential pathological changes.

### Statistical analysis

All data were calculated as mean ± standard deviation. Statistical analysis was performed using one-way analysis of variance or student's t-test to compare differences between groups. A p-value of less than 0.05 was considered statistically significant. Asterisks indicate *p < 0.05, **p < 0.01, ***p < 0.001.

## Supplementary Material

Supplementary figures and tables.

## Abbreviations

TPE: (4-(1,2,2-Triphenylvinyl)phenyl)methanol
Azo: (4,4'-azo-diphenyl)-di-acetic acid
DiR: 1,1′-Dioctadecyl-3,3,3′,3′ tetramethylindotricarbocyanine iodide
NAs: Nanoassemblies
AIE: Aggregation-induced emission
FBS: Fetal bovine serum
FRET: Förster resonance energy transfer
ACQ: Aggregation-caused quenching
AIEgens: AIE fluorogens
DSPE-PEG_2K_: 1,2-Distearoyl-sn-glycero-3-phosphoethanolamine-N-[methoxy(polyethyleneglycol)-2000]
MCTS: Multicellular tumor spheroid
PBS: Phosphate buffered solution
NaCl: Sodium chloride
SDS: Sodium dodecyl sulfate
4T1: Mouse breast cancer cells
3T3: Mouse embryonic fibroblasts
L02: Normal human liver cells
MCF-7: Human breast cancer cell
IVIS: *In vivo* imaging system
CLSM: Confocal microscopy
EDCI: 1-ethyl-3(3-dimethylpropylamine) carbodiimide
DMAP: 4-dimethylaminopyridine
MTT: 3-(4,5-dimthyl-2-thiazolyl)-2,5-dipphenyl-2H-terazolium bromide
HOBT: 1-Hydroxybenzotriazole
