# Supplementary material for: A FRET-based off-on AIE nanoprobe enables instant and stain-free detection of hypoxic niches in tumor sections
Source: Theranostics. 2025 Jun 9;15(14):6651–64. doi: 10.7150/thno.113038 (PMC12203678; doi:10.7150/thno.113038)
Supplement: Supplementary file 1 — Supplementary figures and tables. [file thnov15p6651s1.pdf]

Supplementary materials

A FRET-based off-on AIE nanoprobe enables instant and stain-free detection of hypoxic niches in tumor sections

Authors: Chen Wang[1], Jun Shen[1], Muredili Muhetaer[1], Shenwu Zhang[1], Jin Sun[1,2], Zhonggui He[1,2], Yuequan Wang[1*], Cong Luo[1,2*]

Affiliations:

[1]Department of Pharmaceutics, Wuya College of Innovation, Shenyang Pharmaceutical University, Shenyang 110016, PR China

[2]Joint International Research Laboratory of Intelligent Drug Delivery Systems of Ministry of Education, Shenyang Pharmaceutical University, Shenyang 110016, China

***Corresponding authors:**

Yuequan Wang, Ph.D.; Cong Luo, Ph.D.

Department of Pharmaceutics, Wuya College of Innovation, Shenyang Pharmaceutical University, 103 Wenhua Road, Shenyang 110016, China

Tel: +86-024-23986321; Fax: +86-024-23986321

E-mail address: wangyuequan@aliyun.com; luocong@syphu.edu.cn

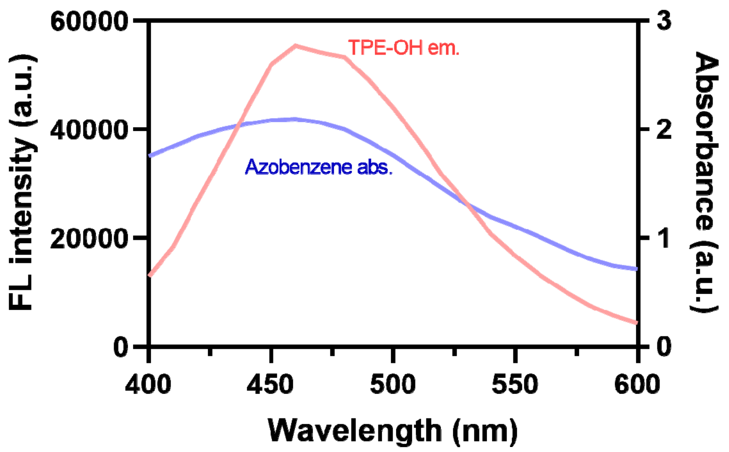

Figure S1. Fluorescence spectrum of TPE and absorption spectrum of Azo.

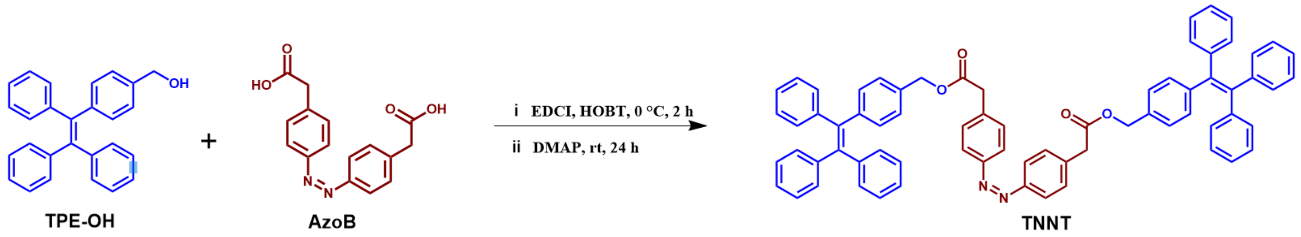

Figure S2. Synthesis route of TNNT.

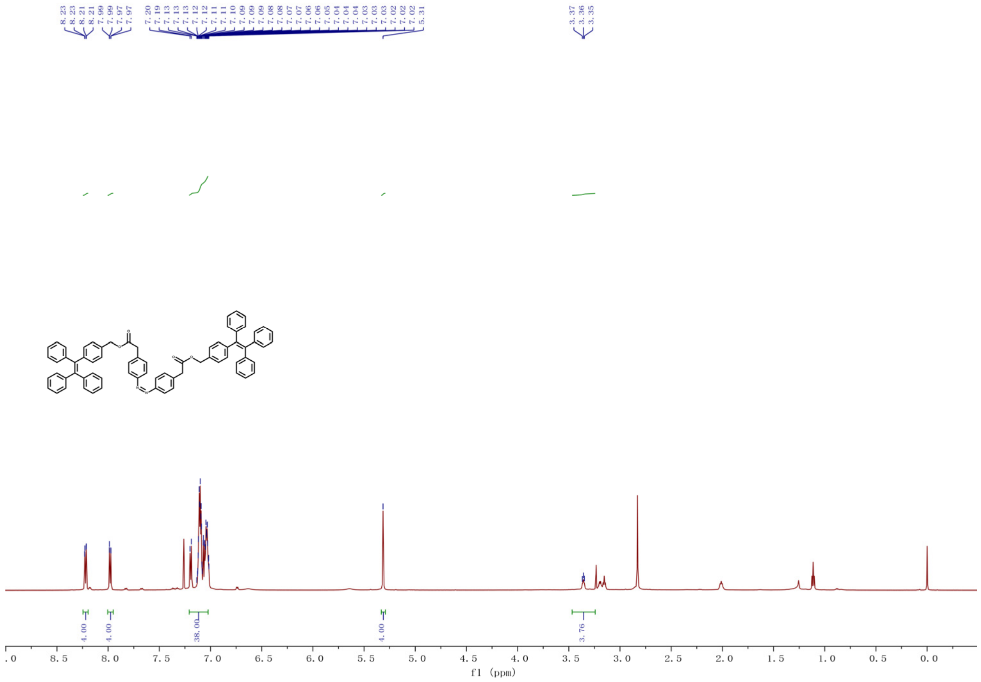

Figure S3. ^{1}H NMR spectra of TNNT. ^{1}H NMR (600 MHz, $CDCl_3$) δ = 8.22 (dd, J = 8.4, 1.3, 4H), 8.00-7.95 (m, 4H), 7.21-7.02 (m, 38H), 5.31 (s, 4H), 3.36 (t, J = 6.0, 4H).

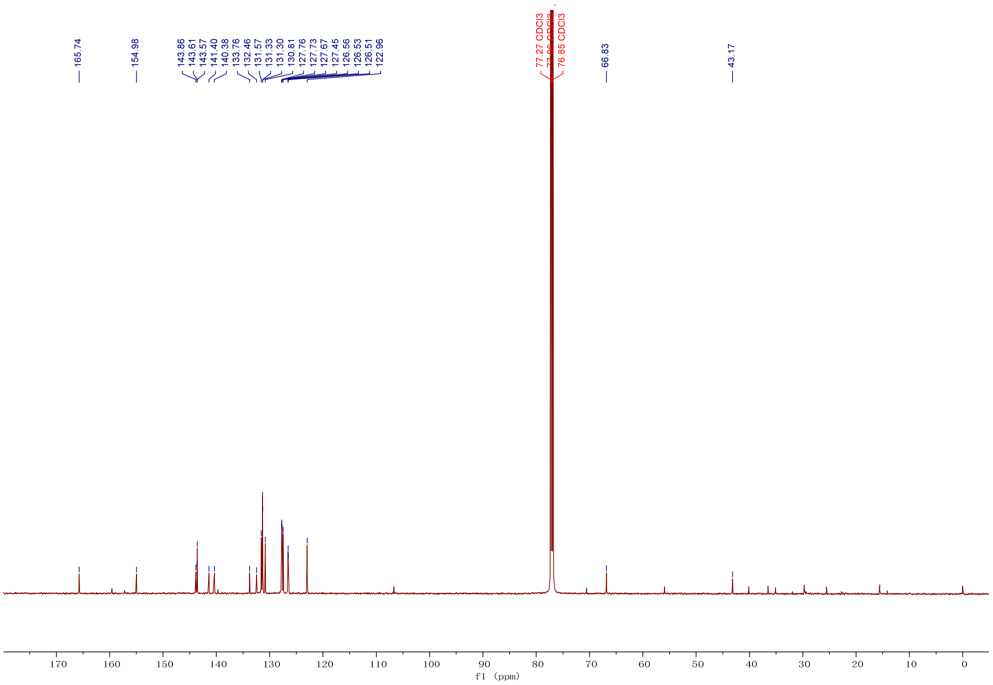

Figure S4. ^{13}C NMR (151 MHz, Chloroform-d) δ = 165.74, 154.98, 143.86, 143.57, 141.40, 140.38, 133.76, 131.57, 131.33, 131.30, 130.81, 127.76, 127.73, 127.67, 127.45, 126.56, 126.53, 126.51, 122.96, 66.83, 43.17.

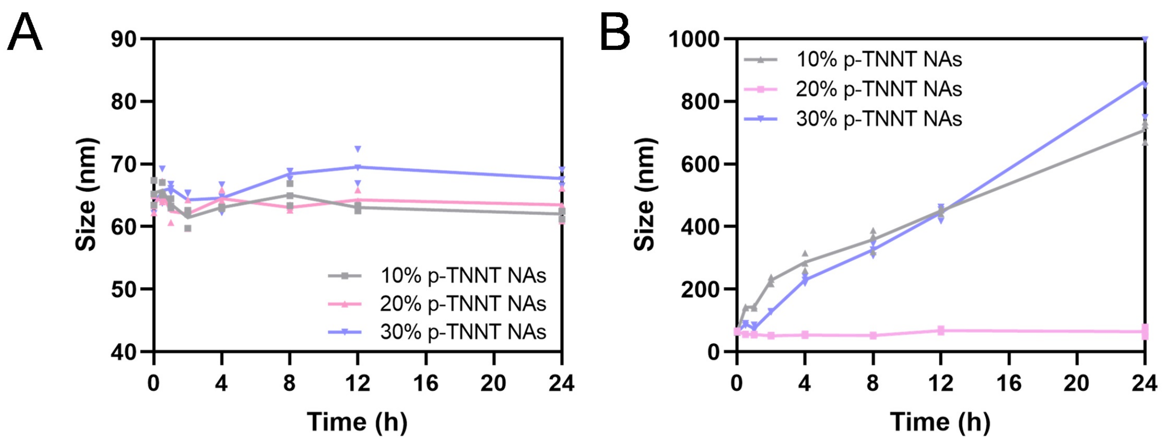

Figure S5. Colloidal stability of the PEGylated TNNT NAs with different proportions of DSPE-PEG_{2K} incubated in (A) PBS (pH 7.4) and (B) PBS (pH 7.4) with 10% FBS in a 37°C shake table under dark conditions (n = 3).

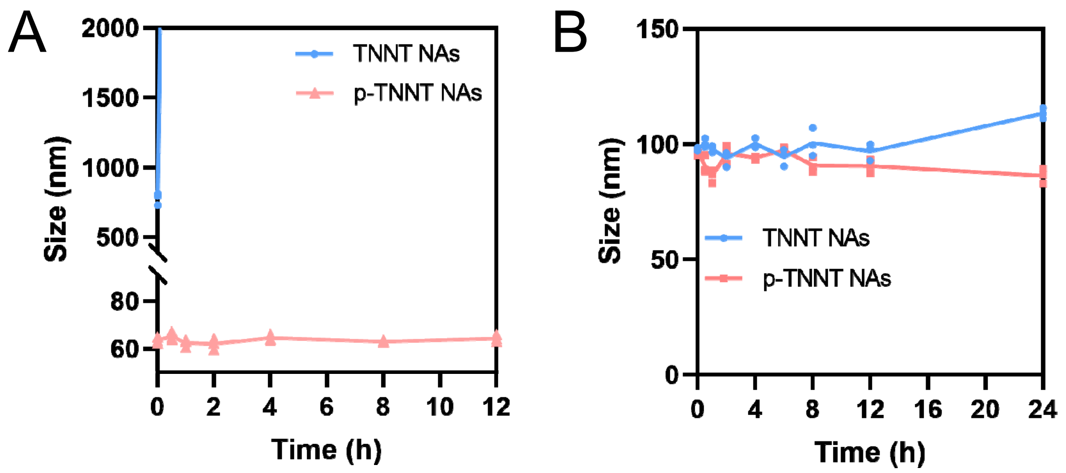

Figure S6. Colloidal stability of NAs incubated in (A) PBS (pH 7.4) and (B) PBS (pH 7.4) with 10% plasma of Sprague Dawley rats in a 37°C shake table under dark conditions (n = 3).

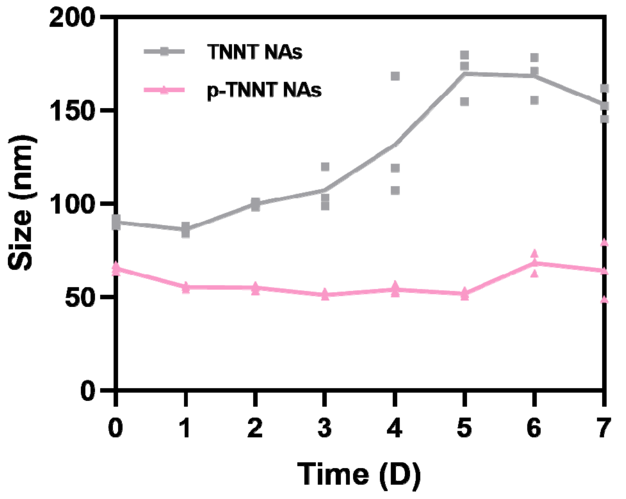

Figure S7. Storage stability of TNNT NAs and p-TNNT NAs stored at 4°C.

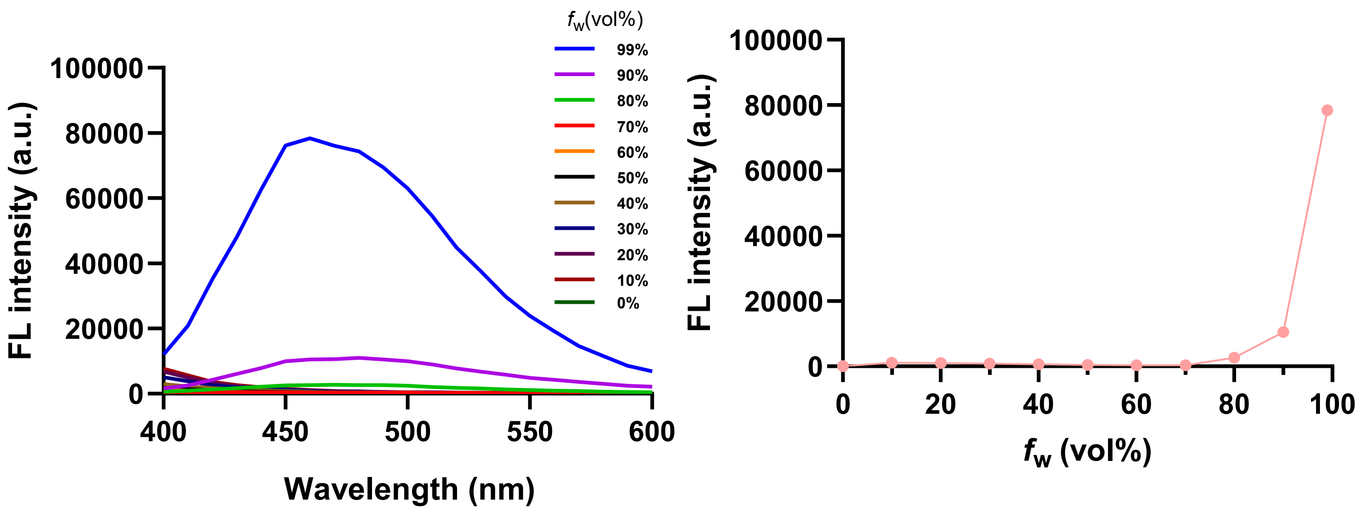

Figure S8. Fluorescence spectrum of TPE in THF/water mixtures with different water fractions (fw). λex = 320 nm.

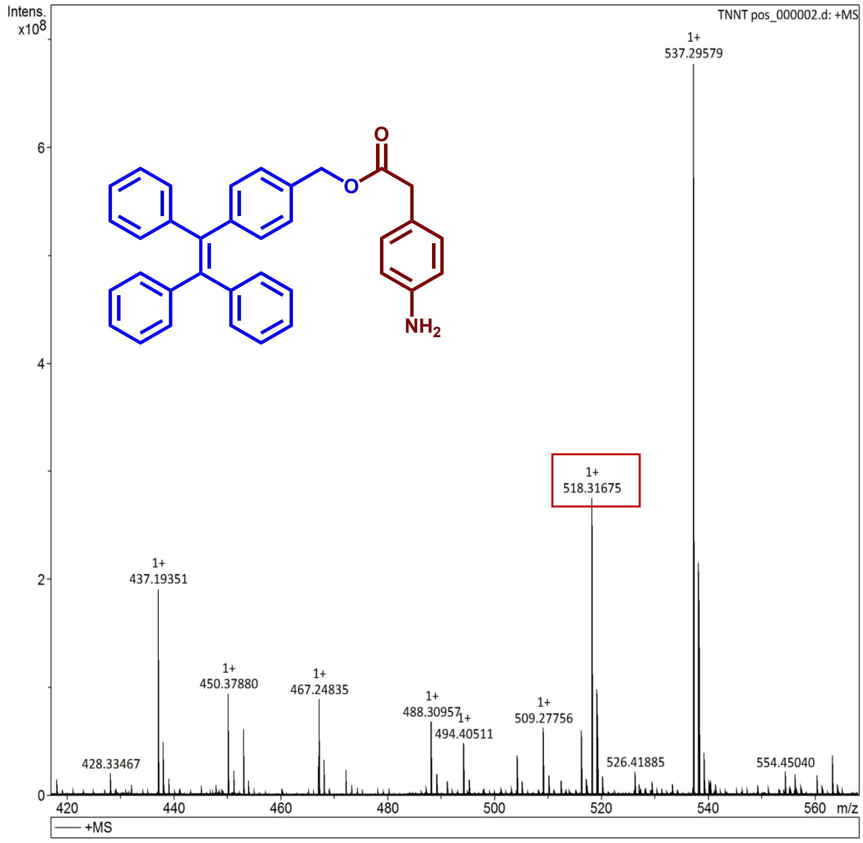

Figure S9. Mass spectrometry result of TPE-NH_2.

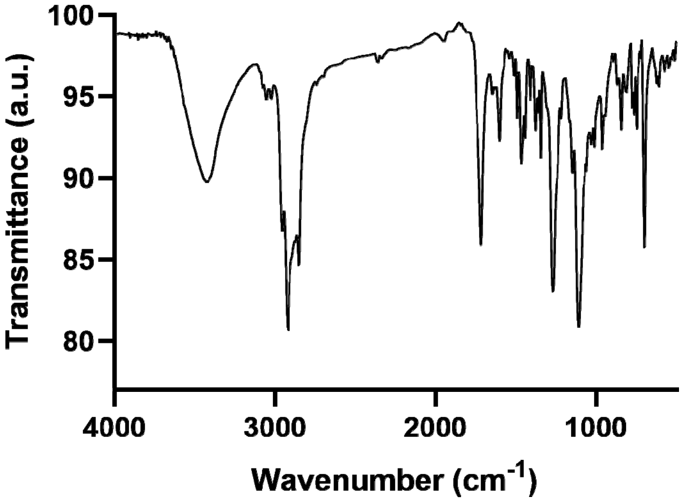

Figure S10. FTIR of TPE-NH_2.

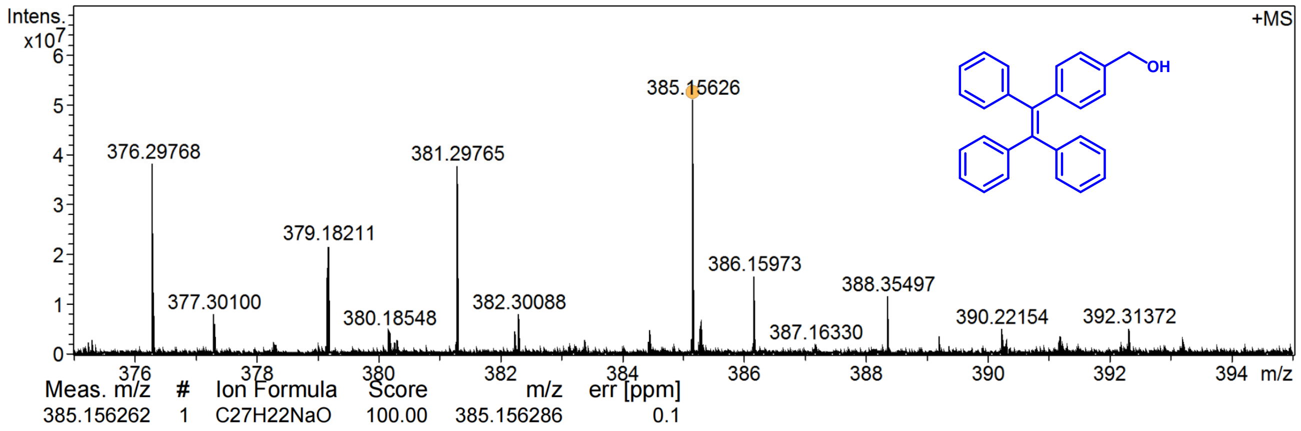

Figure S11. Mass spectrometry result of TPE.

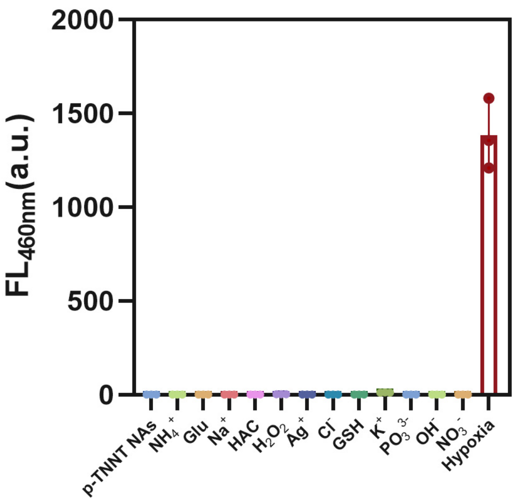

Figure S12. Fluorescence intensity under different physiological conditions of the p-TNNT NAs.

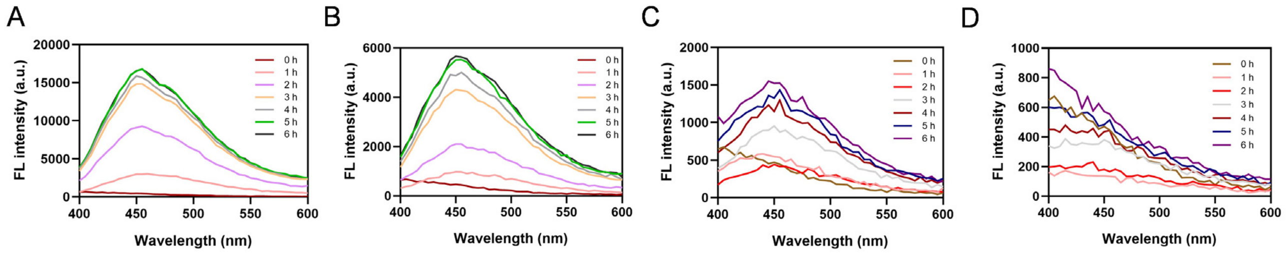

Figure S13. Fluorescence recovery of nanoprobe at 0.1、0.05、0.02、0.005 mg/mL in sodium dithionite solution.

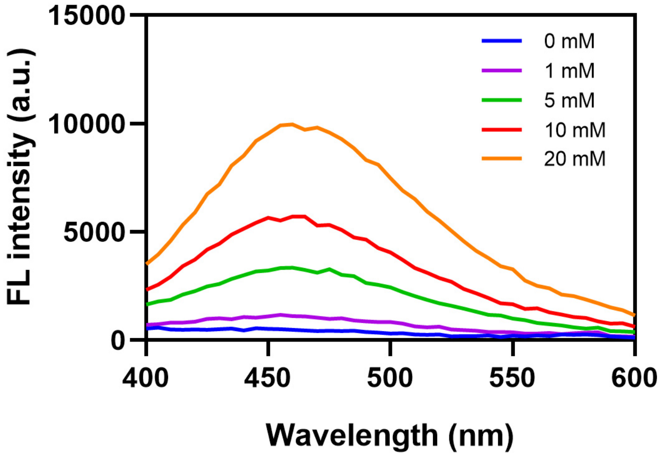

Figure S14. Fluorescence recovery of nanoprobe in different concentrations sodium dithionite solution.

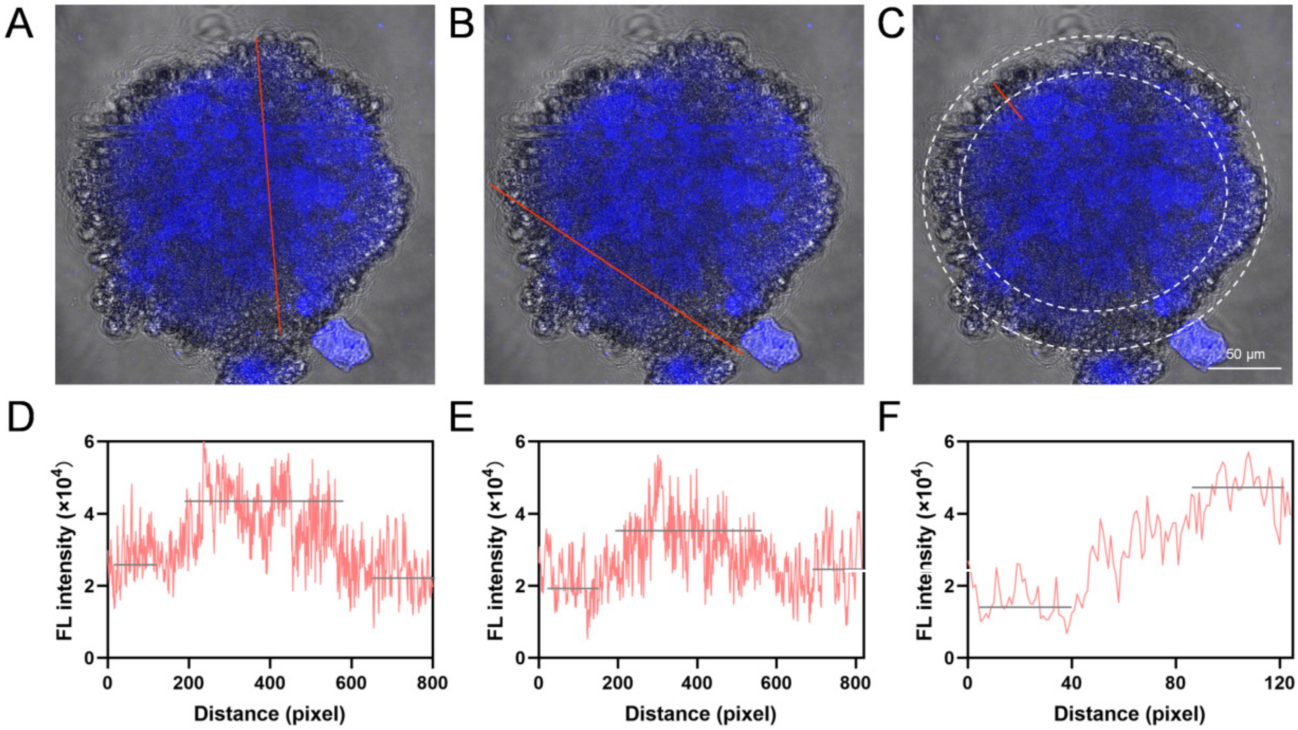

Figure S15. (A-C) The CLSM images of p-TNNT NAs in 4T1 multicellular tumor spheroids at a depth of 50 μm. Scale bar: 50 μm. (D-F) Fluorescence quantitative analysis of images in A-C red line.

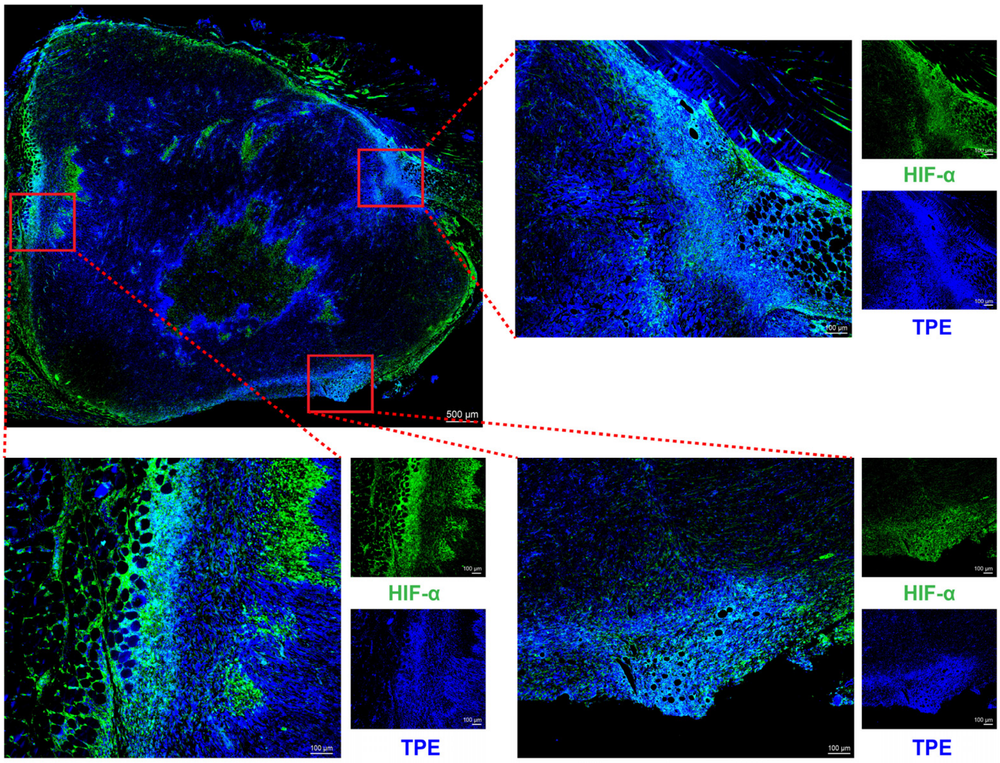

Figure S16. The immunofluorescent staining of tumor slices after treated with p-TNNT NAs and HIF-α. (A) Scale bar: 500 μm. (B) Scale bar: 100 μm.

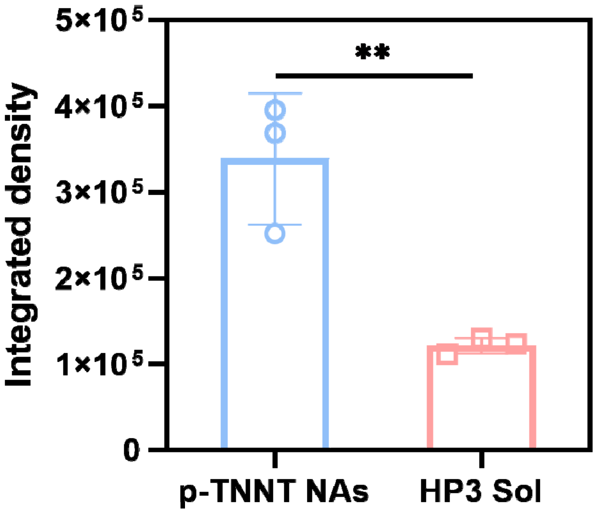

Figure S17. Fluorescence semiquantitative analysis of Figure 5G by Image J (n = 3).

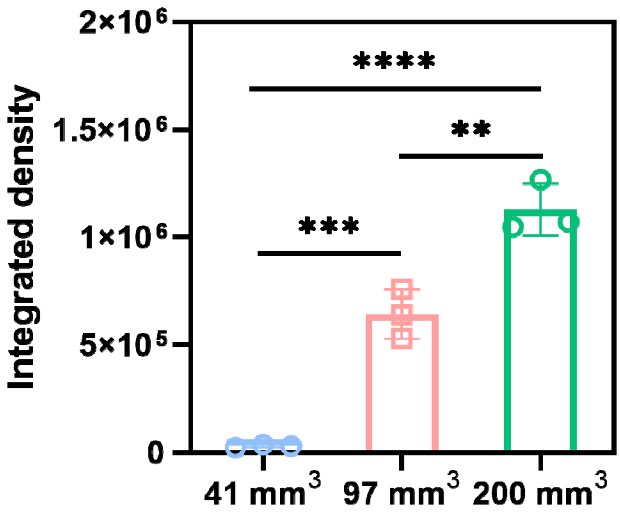

Figure S18. Fluorescence semiquantitative analysis of Figure 5H by Image J (n = 3).

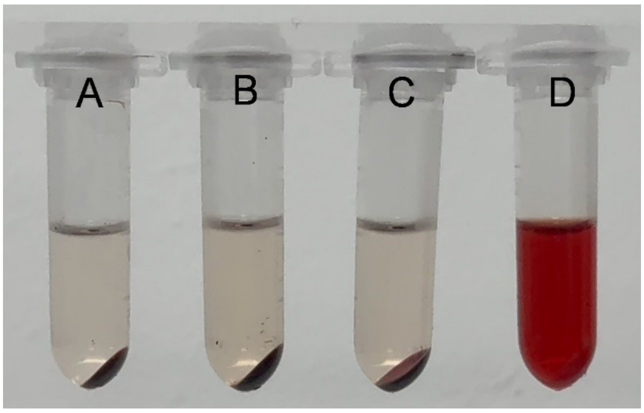

Figure S19. Hemolysis photograph. A: Pure water. B: TNNT NAs. C: p-TNNT NAs. D: Saline.

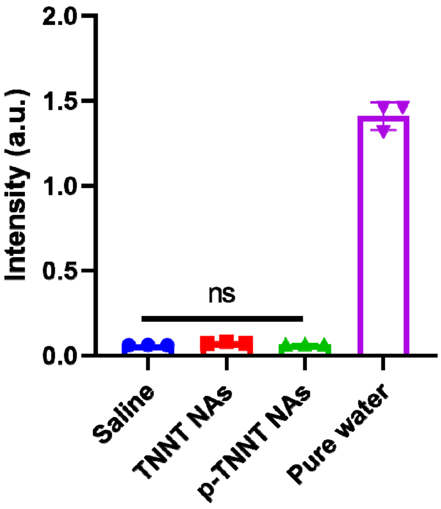

Figure S20. Quantitative analysis of hemoglobin content in the supernatants (n = 3).

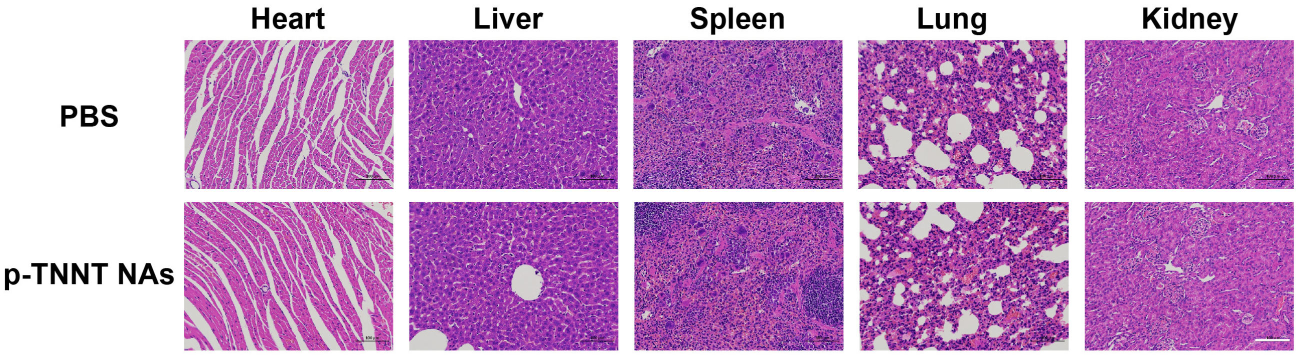

Figure S21. Sections of tumor tissue were stained with CD31 to stain blood vessels, PI to stain nuclei, and TNNT to characterize hypoxic sites. The scale bar represents 50 μm.

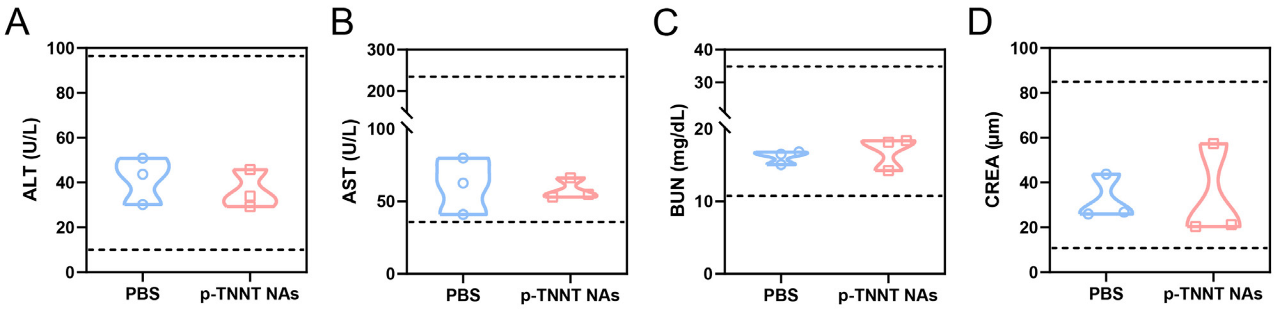

Figure S22. Hepatorenal function parameters after different treatments (n = 3).

Table S1. Characterization of the PEGylated TNNT NAs with different proportions of DSPE-PEG_{2K} (n = 3).

Formulations	Size (nm)	PDI
10%	65.30 ± 1.98	0.13 ± 0.05
20%	63.50 ± 1.36	0.14 ± 0.04
30%	63.40 ± 1.35	0.12 ± 0.04

Table S2. Characterization of TNNT NAs and p-TNNT NAs (n = 3).

**Nanoassemblies**	**Size (nm)**	**PDI**	**Zeta (mV)**
TNNT NAs	118.40 ± 1.30	0.15 ± 0.02	-17.37 ± 5.25
p-TNNT NAs	63.50 ± 1.36	0.14 ± 0.04	-22.83 ± 2.04

Table S3. Pharmacokinetic parameters of DiR Sol and DiR/p-TNNT NAs (n = 3).

**Formulations**	[a] **$AUC_{0\text{-}24\ h}$**	[b] **$C_{0.5}$**
DiR Sol	0.96 ± 0.19	0.04 ± 0.03
DiR/p-TNNT NAs	99.98 ± 6.07	10.97 ± 0.67

[a] Area under the plasma concentration-time curve (nmol/mL*h). **[b]** The plasma concentration at 0.5 h time point (nmol mL^{-1}).

Table S4. The length, width and volume of the tumors.

Group	Length (mm)	Width (mm)	Volume (mm^3)
1	7.40	3.34	41.28
2	5.85	5.77	97.38
3	7.66	7.24	200.76
